# Impact of SARS-CoV-2 vaccination and of seasonal variations on the innate immune inflammatory response

**DOI:** 10.3389/fimmu.2024.1513717

**Published:** 2025-01-14

**Authors:** Hend Jarras, Isalie Blais, Benjamin Goyer, Wilfried W. Bazié, Henintsoa Rabezanahary, Mathieu Thériault, Kim Santerre, Marc-André Langlois, Jean-François Masson, Joelle N. Pelletier, Nicholas Brousseau, Denis Boudreau, Sylvie Trottier, Mariana Baz, Caroline Gilbert

**Affiliations:** ^1^ Axe de Recherche Maladies Infectieuses et Immunitaires, Centre de Recherche du CHU de Québec-Université Laval, Quebec City, QC, Canada; ^2^ Programme de Recherche sur les Maladies Infectieuses, Centre Muraz, Institut National de Santé Publique, Bobo-Dioulasso, Houet, Burkina Faso; ^3^ Department of Biochemistry, Microbiology and Immunology, Faculty of Medicine, University of Ottawa, Ottawa, ON, Canada; ^4^ Department of Chemistry, Institut Courtois, Quebec Center for Advanced Materials, Regroupement québécois sur les matériaux de pointe, and Centre Interdisciplinaire de Recherche sur le Cerveau et l’Apprentissage, Université de Montréal, Montreal, QC, Canada; ^5^ Department of Chemistry, Department of Biochemistry, Université de Montréal, Montreal, QC, Canada; ^6^ PROTEO — The Québec Network for Research on Protein Function, Engineering, and Applications, Quebec City, QC, Canada; ^7^ Biological Risks Department, Institut national de santé publique du Québec, Quebec City, QC, Canada; ^8^ Département de Chimie et Center for Optics, Photonics and Lasers (COPL), Université Laval, Quebec City, QC, Canada; ^9^ Département de Microbiologie-Infectiologie et d’Immunologie, Faculté de Médecine, Université Laval, Quebec City, QC, Canada

**Keywords:** SARS-CoV-2, vaccine, season, innate immunity, peripheral blood mononuclear cells, polymorphonuclear neutrophils, TLR7/8

## Abstract

**Introduction:**

The innate immune response is an important first checkpoint in the evolution of an infection. Although adaptive immunity is generally considered the immune component that retains antigenic memory, innate immune responses can also be affected by previous stimulations. This study evaluated the impact of vaccination on innate cell activation by TLR7/8 agonist R848, as well as seasonal variations.

**Methods:**

To this end, blood samples from a cohort of 304 food and retail workers from the Quebec City region were collected during three visits at 12-week intervals. Peripheral blood mononuclear cells and polymorphonuclear neutrophils were isolated during the first and third visits and were stimulated with R848 to assess the innate immune response.

**Results:**

Our results show that IL-8 production after stimulation decreased after vaccination. In addition, the IL-8 response was significantly different depending on the season when the visit occurred, for both COVID-19 vaccinated and unvaccinated individuals.

**Discussion:**

This study highlights that innate immune responses can be affected by SARS-CoV-2 vaccination and fluctuate seasonally.

## 1 Introduction

The emergence of the novel severe acute respiratory syndrome coronavirus 2 (SARS-CoV-2) in late 2019 and its rapid spread causing a pandemic has given many challenges to the immune field (1). While most patients developed mild symptoms, a significant proportion of cases progressed into a more severe illness (2). The ability of the immune system to recognize the virus and mount an efficient response is thus crucial to avoid an unfavorable disease course. Innate immune cells are the first to sense viral infection through pattern recognition receptors (PRRs) (3, 4). This results in the expression of type I and type III interferons (IFN) that induce an antiviral response, as well as the secretion of pro-inflammatory cytokines (4–7).

Toll-like receptors (TLRs) are a class of PRRs, of which TLR3, TLR7, TLR8 and TLR9 recognize various types of nucleic acids (8, 9). TLR7 and TLR8, in particular, detect ssRNA and have been found to play an important role in the response against SARS-CoV-2 (7, 8, 10). In comparison to common coronaviruses, SARS-CoV-2 has specific pathogen-associated molecular patterns (PAMPs), such as a four-amino-acid insertion (PRRA) in its spike protein and a higher density of GU-rich fragments capable of activating TLR7/8 (11). It is also known that those receptors can be differentially activated by different RNA sequences and immune modifiers such as R848 (12, 13). Furthermore, this is a good agonist to study the impact of SARS-CoV-2 nucleic acids on innate cells (7, 14). Peripheral blood mononuclear cells (PBMCs) express both TLR7 and TLR8, while polymorphonuclear neutrophils (PMNs) express only TLR8 (15, 16). Additionally, SARS-CoV-2 variants exhibit varying capacities to activate neutrophils via TLR8, with the Delta variant causing stronger activation than the initial strain, while the Omicron variant induces weaker activation (17). R848 can be used to activate neutrophils, monocytes and other immune cells, resulting in the production of cytokines such as interleukin-8 (IL-8) (14, 16, 18–24). One of the main functions of IL-8 is to attract and activate neutrophils to the site of inflammation. Neutrophils are the first cells to be recruited to infection sites and therefore are critical to the early immune response (14, 25). Regarding COVID-19, studies have shown that plasma IL-8 levels correlate with disease severity (26, 27). IL-8 released from both PBMCs and PMNs stimulated by R848 is therefore a good indicator of innate immune activation.

Following the initiation of the COVID-19 vaccination campaigns in late 2020, it was established that the approved vaccines elicited a good adaptive, notably humoral, response against the virus in the weeks and months following full vaccination (28–30). Although later studies showed that circulating antibody levels rapidly decrease over time, cellular immunity tends to persist longer and is thought to protect against severe forms of the disease (31–36). However, the effect of vaccines on innate immunity has not been thoroughly explored. It is known that SARS-CoV-2 acts on innate immune cells to delay their response, notably by inhibiting the initial IFN response (5, 37). Moreover, changes in innate immune cells have been detected in recovered COVID-19 patients (38). It is thus of interest to study the innate immune response in relation to COVID-19 vaccination.

Furthermore, the peaks of SARS-CoV-2 infections varied seasonally in the province of Quebec. Indeed, from the onset of the pandemic until the emergence of Omicron variants, peaks of infection happened mostly in fall, winter and spring, decreasing significantly in summer (39). It is additionally established that other respiratory viruses, such as influenza, peak in winter in the province of Quebec (40). It is therefore relevant to also study the impact of seasonality on the immune response.

This study aims to explore the role of vaccination and the seasonal variation on the innate immune system following activation. This work is part of a larger longitudinal study conducted on food and retail workers in the Quebec City metropolitan area to determine the rate of infection and to examine the immune response to SARS-CoV-2. The distinctive capacity of SARS-CoV-2 to activate TLRs, compared to common coronaviruses, has guided the design of the immune response test, by including a dose-response of TLR7/8 agonist to stimulate two major types of leukocytes, mono (PBMCs) and polymorphonuclear (PMNs) cells to produce IL-8.

## 2 Methods

### 2.1 Study participants

**Figure 1:**
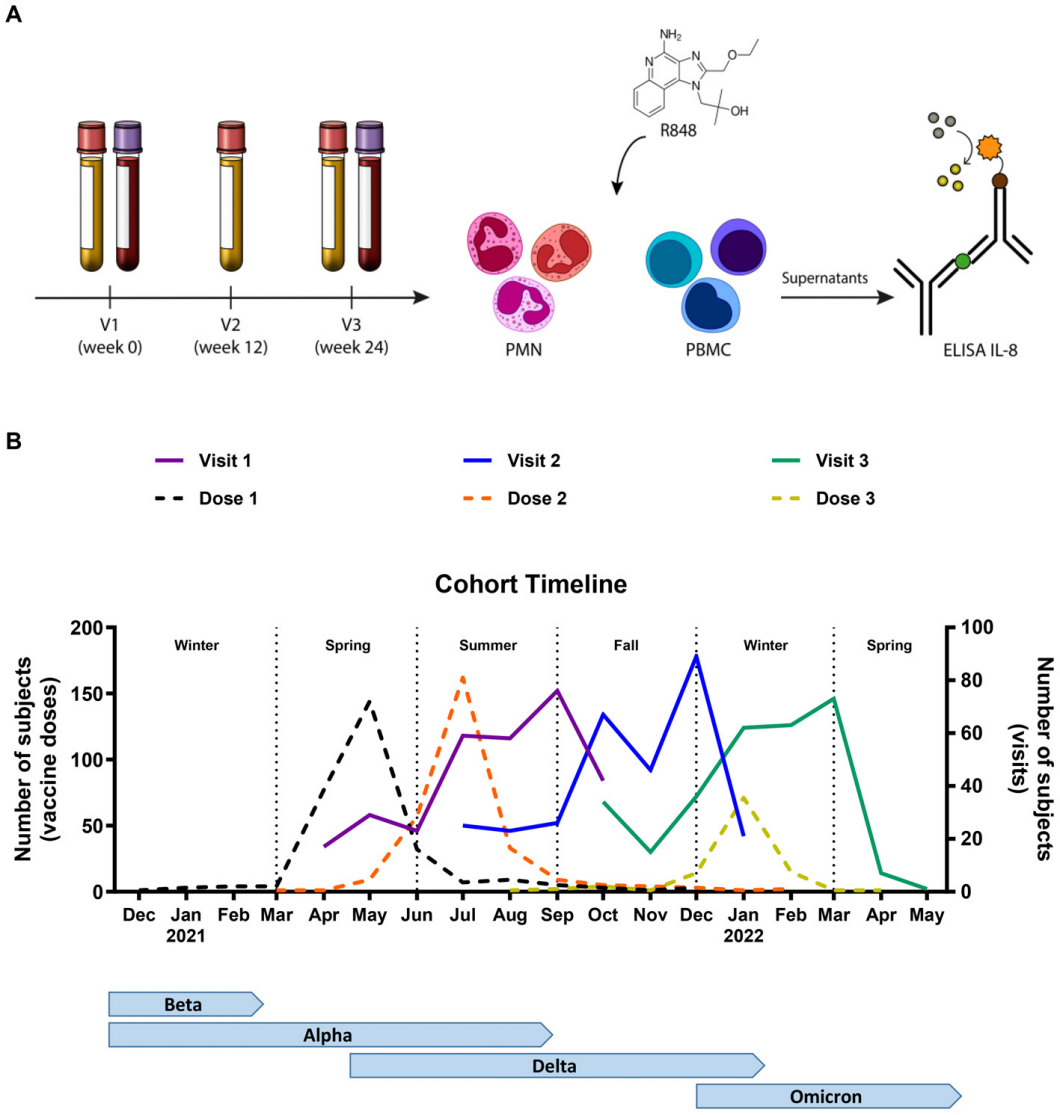
Timeline of the study. **(A)** Schematic representation of the methodology. Participants came in for three visits (V), and whole blood was collected at V1 and V3. PMNs and PBMCs were isolated and stimulated with R848 for 24 h. IL-8 ELISA was conducted on the cell culture supernatants. **(B)** Representation of the number of participants at each visit (full line), as well as the vaccine doses received (dotted line) per month in relation to the seasons and the circulating variants of concern (VOC) in the province of Quebec. V1 n=304; V2 n=297; V3 n=291; Dose 1 n=293; Dose 2 n=287; Dose 3 n=110.

Participants for this study were recruited at the *“Centre Hospitalier Universitaire de Québec – Université Laval (CHUL)”* in Quebec City, Canada, as part of a longitudinal study of the humoral and cellular responses to SARS-CoV-2 (41, 42). The cohort consisted of 304 adult volunteers over 18 years of age who were food and retail workers (bar/restaurant, grocery, or hardware store) from the greater Quebec City area at the time of recruitment. Participants were enrolled after giving written informed consent. They came for three visits at 12-week intervals between April 2021 and May 2022, during which they were surveyed about demographic, socioeconomic, behavioural, and occupational characteristics. Whole blood for immune response analysis was collected at visit one (V1) and visit three (V3) (**Figure 1A**). The study was approved by the “*Comité d’éthique de la recherche du CHU de Québec – Université Laval*” (registration number 2021-5744).

### 2.2 Neutrophil purification

Peripheral blood from venipuncture was collected in EDTA-containing tubes and processed within two hours. PMNs were isolated as previously described (43). In brief, 2% dextran (Sigma, Cat. 31392-50G) sedimentation of erythrocytes was first carried out, followed by Lymphocyte Separation Medium (Wisent, Cat. 305-010-CL) density gradient centrifugation. After removing the supernatant, remaining contaminating erythrocytes were eliminated through hypotonic lysis. Resulting purified granulocytes were then used for R848 (Resiquimod) (InvivoGen, Cat. vac-r848) stimulation.

### 2.3 PBMC purification

Peripheral blood was first centrifuged to remove plasma. PBMCs were isolated as previously described (44). Briefly, they were isolated using Lymphocyte Separation Medium (Wisent, Cat. 305-010-CL) density gradient centrifugation. The lymphocyte ring was collected and washed. PBMCs were then used for R848 (InvivoGen, Cat. vac-r848) stimulation.

### 2.4 TLR7/8 stimulation

After isolation from blood samples, PBMCs and PMNs were resuspended at a concentration of 2 x 10^6^ cells/mL in the high-performance TheraPEAK™ X-VIVO™-15 Serum-free Hematopoietic Cell Medium (Lonza, Cat. BEBP04-744Q). They were plated in round-bottom 96-well plates at 1 x 10^5^ cells/well and 2 x 10^5^ cells/well and stimulated with the viral analogue R848 (InvivoGen, Cat. vac-r848). Four R848 conditions (0, 0.5, 1 and 2 µg/mL) were analyzed in triplicate. They were then incubated for 24 h at 37°C, 5% CO_2_. The next day, plates were centrifuged 5 min at 400 x *g* and the supernatant was transferred to a flat-bottom 96-well plate and frozen at -20°C until use.

### 2.5 ELISA

The human IL-8/CXCL8 Duoset ELISA kit from R&D Systems (Cat. DY208) was used to measure IL-8 concentrations in the supernatants of R848-stimulated cells. Ninety-six-well plates were coated with 4 µg/mL of diluted mouse anti-human capture antibody overnight at room temperature. The next morning, plates were rinsed three times with wash buffer (0.05% Tween-20 in phosphate-buffered saline (PBS)) with the HydroControl washer (Tecan, Salzburg, Austria) and incubated for 1 h with block buffer (1% bovine serum albumin (BSA) in PBS, pH 7.2-7.4, 0.2 µm filtered, R&D Systems, Cat. DY995) at room temperature. After rinsing, 100 µL of samples, standards (31.3-2000 µg/mL) and controls were added and incubated for 2 h at room temperature. Plates were washed, then 10 ng/mL of biotinylated goat anti-human detection antibody was added to the wells for 2 h at room temperature. After washing, streptavidin-HRP diluted 40-fold was added and left to incubate for 20 min at room temperature in the dark. Plates were washed again and prepared for the detection of bound antibodies by adding the substrate solution (1:1 mixture of H_2_O_2_ and tetramethylbenzidine, R&D Systems, Cat. DY999) for 20 min at room temperature in the dark. The stop solution (2N H_2_SO_4_, R&D Systems, Cat. DY994) was added to the wells before reading at 450 and 540 nm with the SpectraMax 190 microplate reader (Molecular Devices, San Jose, CA, USA).

### 2.6 Statistical analysis

All analyses were performed with GraphPad Prism 10.2.0 (GraphPad Inc, San Diego, CA, USA). For all data, initial tests of normality and log normality showed that the data did not fit both distributions. Therefore, all analyses were conducted using nonparametric tests. Where applicable, Friedman or Kruskal-Wallis tests were performed followed by Dunn’s multiple comparisons test for paired or unpaired data, respectively. All tests were two-sided and P-values < 0.05 were considered statistically significant. Data are shown as mean ± standard error of the mean (SEM).

## 3 Results

### 3.1 Cohort description and study timeline

The cohort and study timeline have been described in detail by Santerre et al. (42). Briefly, 304 food and retail workers from the Quebec City metropolitan area, Canada, were enrolled in order to study innate and adaptive immunity to SARS-CoV-2 (**Table 1**). Their occupational distribution was as follows: 149 bar/restaurant workers (49%), 112 grocery store employees (37%), and 43 hardware store workers (14%). Men accounted for 42% of participants (n=128) and women for 58% (n=176). As for age, 139 participants were between 18 and 39 years old (46%), 118 were between 40 and 59 (39%), and 47 were 60 years or older (15%). The median age was 41 years old (interquartile range (IQR) 26-56). Three visits were planned for each participant at 12-week intervals. The first visit (V1) took place between April and October 2021, the second visit (V2) between July 2021 and January 2022, and the third visit (V3) between October 2021 and May 2022 (**Figure 1B**). The first vaccination campaign had already begun prior to recruitment. Thus, before V1, most participants had received at least one dose of vaccine (79%) and 63% were already fully vaccinated (two or more vaccine doses). Many participants (38%) also received a third dose before their third visit, which mostly coincided with the fifth wave (emergence of the Omicron variant) of the pandemic.

**Table 1:** Demographic and clinical characteristics of study participants.

	Participants n=304
Occupation, n (%)	
Restaurant/Bar	149 (49.0)
Grocery	112 (36.8)
Hardware	43 (14.1)
Sex, n (%)	
Men	128 (42.1)
Women	176 (57.9)
Age, n (%)	
Median (IQR)	41 (26-56)
[18,39]	139 (45.7)
[40-59]	118 (38.8)
[60+]	47 (15.5)
BMI, n (%)	
Median (IQR)	26.1 (22.8-31.1)
< 18.5 (Underweight)	4 (1.3)
18.5-24.9 (Normal weight)	125 (41.1)
25.0-29.9 (Overweight)	82 (27.0)
30.0 (Obese)	93 (30.6)
Comorbidities, n (%)	
At least one	146 (48.0)
Hypertension	39 (12.8)
Diabetes	18 (5.9)
Asthma	31 (10.2)
Chronic lung disease	6 (2.0)
Chronic heart disease	7 (2.3)
Liver disease	2 (0.7)
Cancer	10 (3.3)
Chronic blood disorder	1 (0.3)
Imrnunodepression	7 (2.3)
Chronic neurological disorder	5 ( 1.6)
Other(s)	98 (32.2)

### 3.2 Innate immune response

**Figure 2:**
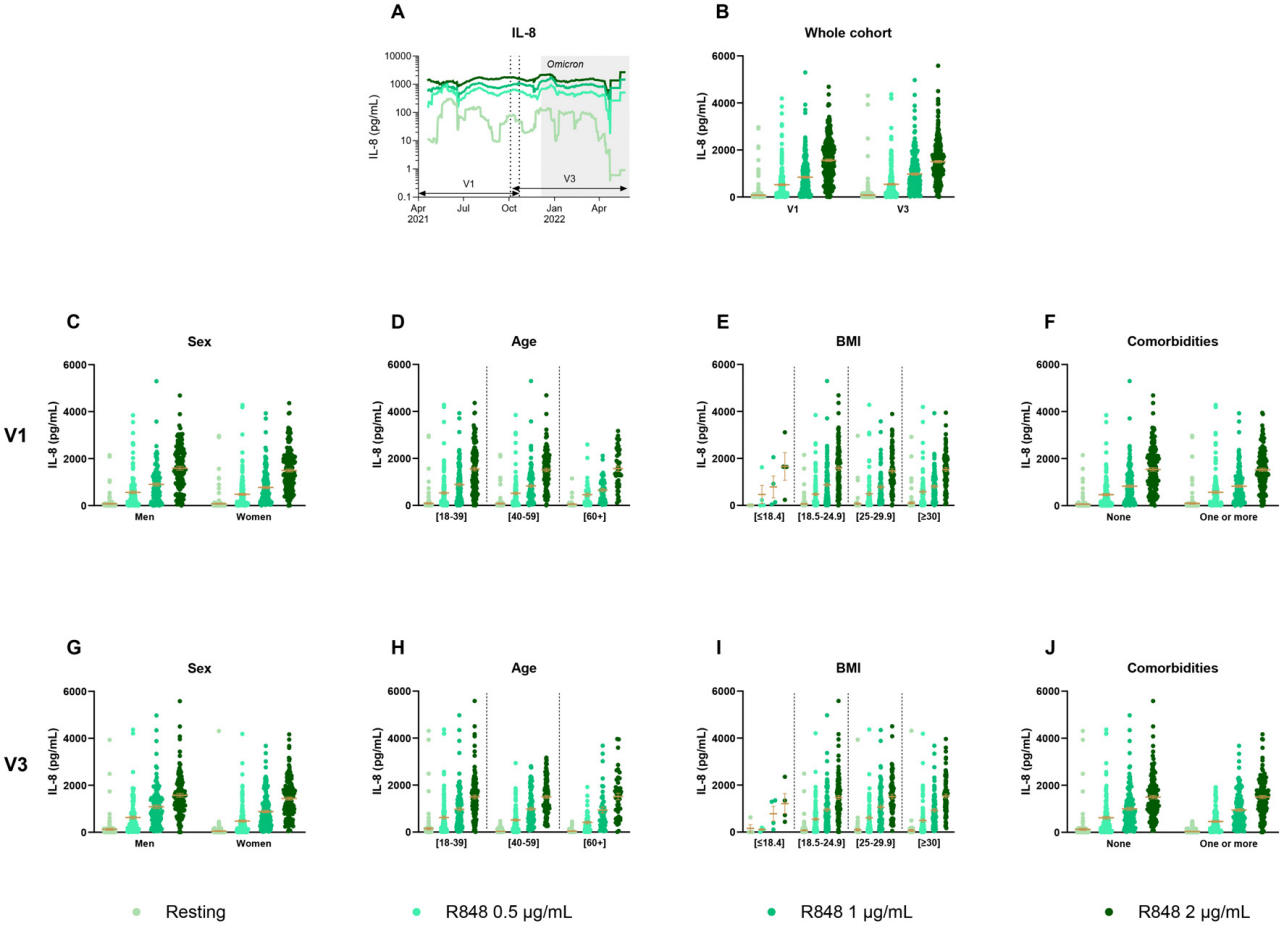
IL-8 production by PMNs separated according to relevant biological data. **(A)** Rolling average of the IL-8 results throughout the study. **(B)** Comparison of the results between visit 1 (V1) and visit 3 (V3) (n=285). **(C)** Comparison of IL-8 production between men (n=128) and women (n=175) at V1. **(D)** Comparison of IL-8 production between different age groups at V1 ([18-39] n=139; [40-59] n=117; [60+] n=47). **(E)** IL-8 measure separated based on BMI group (underweight n=4; normal weight n=121; overweight n=85; obese n=93) at V1. **(F)** IL-8 production at V1 according to the presence of a comorbidity (n=146) or not (n=157). **(G)** Comparison of IL-8 production between men (n=124) and women (n=163) at V3. **(H)** Comparison of IL-8 production between different age groups at V3 ([18-39] n=127; [40-59] n=114; [60+] n=46). **(I)** IL-8 measure separated based on BMI group (underweight n=4; normal weight n=112; overweight n=80; obese n=91) at V3. **(J)** IL-8 production at V3 according to the presence of a comorbidity (n=140) or not (n=147). Data shown are mean ± SEM. Kruskal-Wallis test with Dunn’s multiple comparisons test was used for all graphs except panel **(B)** for which Friedman test was conducted.

**Figure 3:**
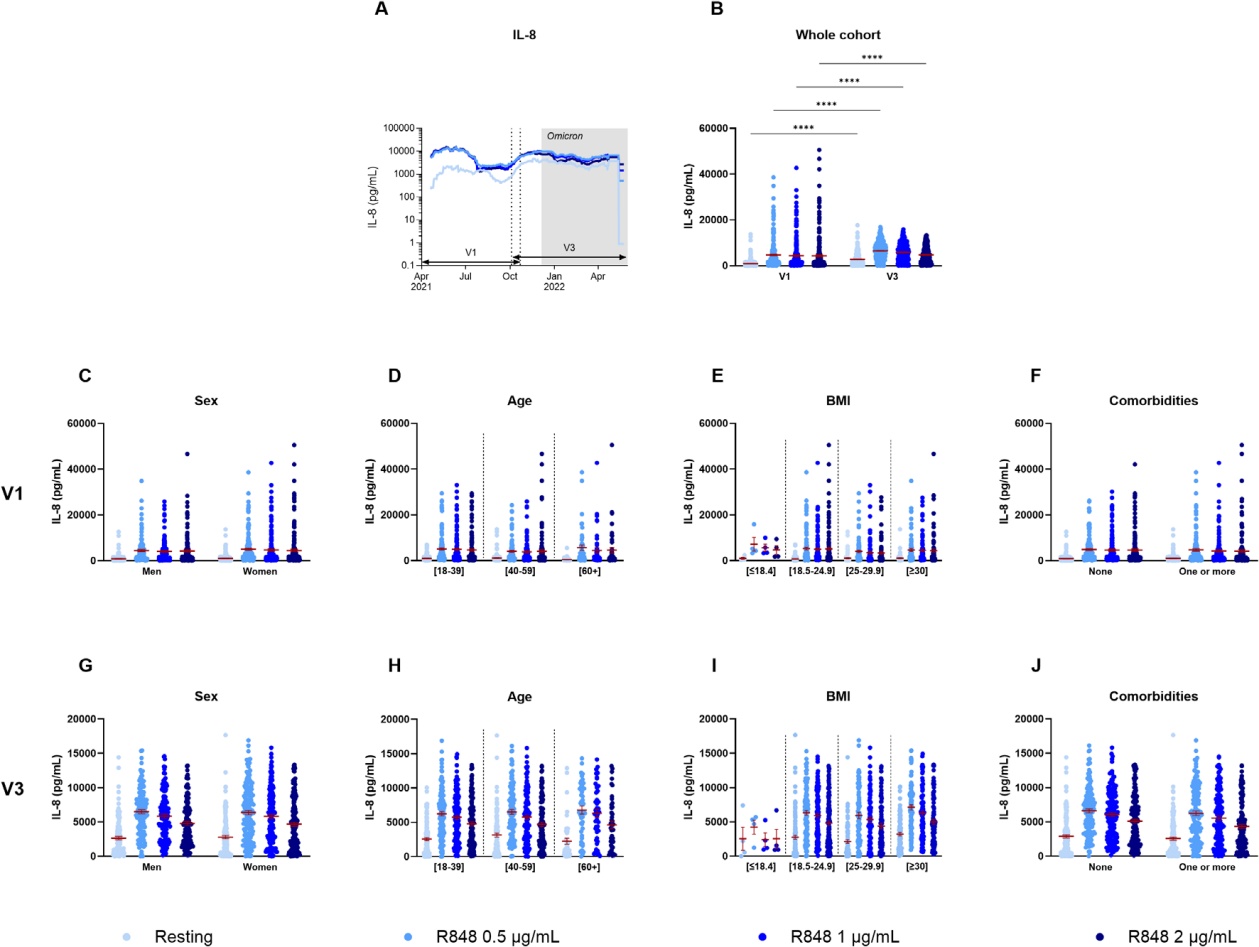
IL-8 production by PBMCs separated according to relevant biological data. **(A)** Rolling average of the IL-8 results throughout the study. **(B)** Comparison of the results between visit 1 (V1) and visit 3 (V3) (n=289). **(C)** Comparison of IL-8 production between men (n=128) and women (n=175) at V1. **(D)** Comparison of IL-8 production between different age groups at V1 ([18-39] n=139; [40-59] n=117; [60+] n=47). **(E)** IL-8 measure separated based on BMI group (underweight n=4; normal weight n=121; overweight n=85; obese n=93) at V1. **(F)** IL-8 production at V1 according to the presence of a comorbidity (n=145) or not (n=158). **(G)** Comparison of IL-8 production between men (n=125) and women (n=165) at V3. **(H)** Comparison of IL-8 production between different age groups at V3 ([18-39] n=131; [40-59] n=114; [60+] n=45). **(I)** IL-8 measure separated based on BMI group (underweight n=4; normal weight n=115; overweight n=80; obese n=91) at V3. **(J)** IL-8 production at V3 according to the presence of a comorbidity (n=141) or not (n=149). Data shown are mean ± SEM. Kruskal-Wallis test with Dunn’s multiple comparisons test was used for all graphs except panel **(B)** for which Friedman test was conducted. **** p < 0.0001.

In order to assess the innate response, IL-8 production by PMNs (**Figure 2**) and PBMCs (**Figure 3**) was first measured following 24 h of stimulation with R848. **Figure 2A** shows a 30-day rolling average of IL-8 production by PMNs to illustrate the global fluctuation over the course of the study. The graph shows that the production increases in a dose-response manner with increasing R848 concentrations. Since there were two time points for each participant, results between both visits were first compared (**Figure 2B**). No significant differences were seen in IL-8 production between V1 and V3 for all conditions. Further analyses were performed using clinical and demographic data such as sex (**Figures 2C, G**), age (**Figures 2D, H**), body mass index (BMI) (**Figures 2E, I**), and the presence of comorbidities (**Figures 2F, J**) at both V1 and V3 separately. Again, results for all of these parameters did not show any significant difference for both visits.

The 30-day rolling average of IL-8 production by PBMCs after R848 stimulation shows some fluctuations throughout the year, yet, unlike PMNs, they do not follow a dose-response pattern (**Figure 3A**). There were also significant differences between both visits for all conditions (**Figure 3B**). Indeed, all values increased at V3 compared to V1, without stimulation and following activation with R848. Again, differences were assessed for sex (**Figures 3C, G**), age (**Figures 3D, H**), BMI (**Figures 3E, I**), and comorbidities (**Figures 3F, J**). There were no significant differences in relation to all those parameters neither at V1 nor at V3.

### 3.3 Impact of vaccination on the inflammatory response

**Figure 4:**
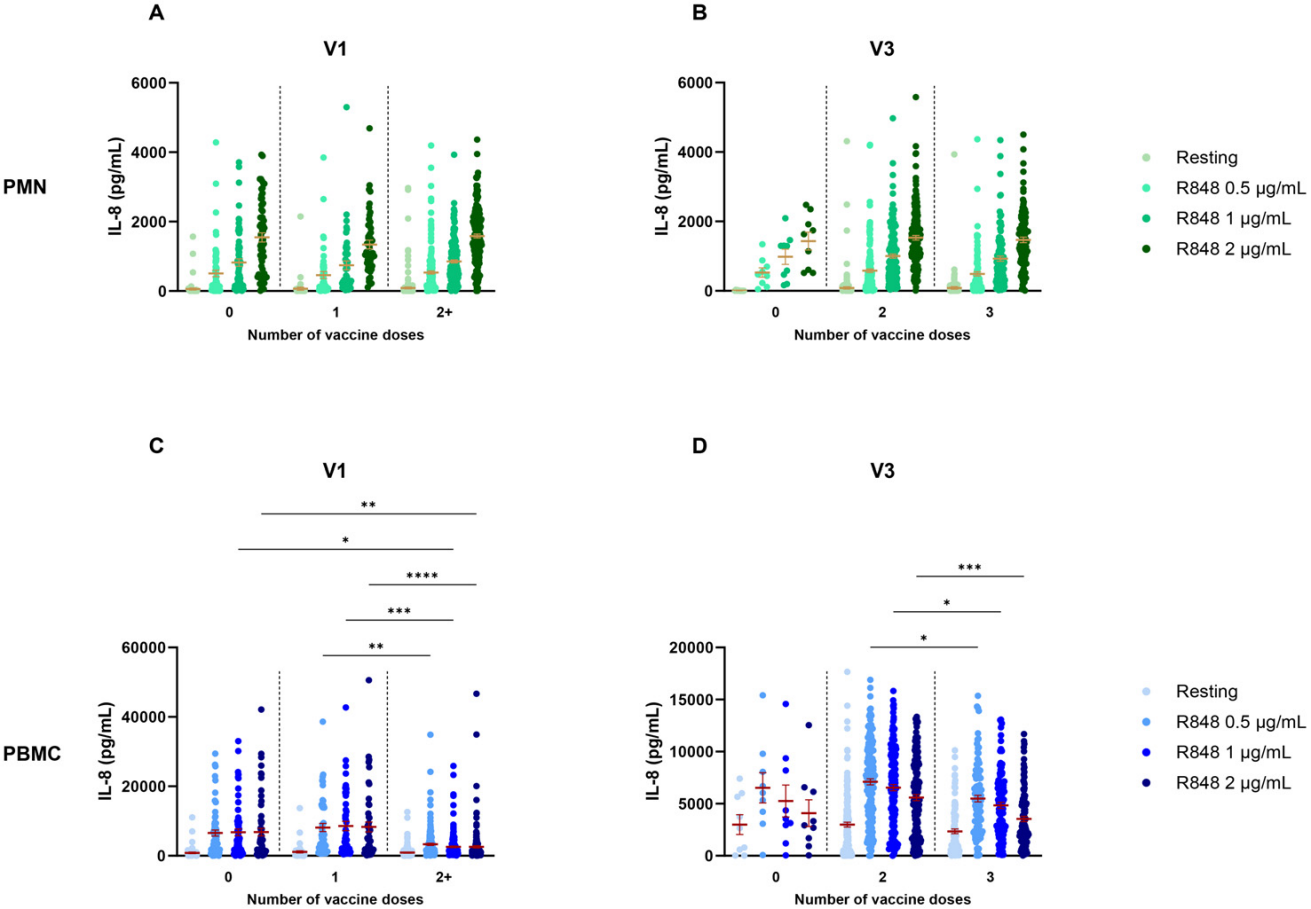
IL-8 response following R848 stimulation separated according to the number of vaccine doses received. **(A)** PMN production of IL-8 at V1 (0 dose n=65; 1 dose n=49; 2 doses n=189). **(B)** IL-8 response by PMNs at V3 (0 dose n=9; 2 doses n=167; 3 doses n=109). **(C)** PBMC IL-8 response at V1 (0 dose n=64; 1 dose n=49; 2 or 3 doses n=190). **(D)** IL-8 production by PBMCs at V3 (0 dose n=9; 2 doses n=169; 3 doses n=110). Data shown are mean ± SEM. Kruskal-Wallis test with Dunn’s multiple comparisons test was used. * p < 0.05; ** p < 0.01; *** p < 0.001, **** p < 0.0001.

Given the information about the vaccine regimen of participants, different parameters were used to analyze IL-8 such as the number of doses received, the type of vaccine and the number of days between the visit and the last dose of vaccine received. Moreover, since previous results showed significant differences between visits, they were analyzed separately for the above-mentioned factors. Regarding the number of vaccine doses received, no differences were found for the PMN response at V1 (**Figure 4A**), nor at V3 (**Figure 4B**). However, results for PBMCs differed between subjects who were fully vaccinated compared to those who were not. Indeed, IL-8 production after R848 stimulation was significantly lower at V1 for fully vaccinated participants compared to unvaccinated and partially vaccinated participants (**Figure 4C**). At V3, subjects who received 3 vaccine doses had a significantly decreased IL-8 response compared to those who got only 2 doses (**Figure 4D**). It is notable that only nine participants remained unvaccinated, and that all other participants were fully vaccinated.

**Figure 5:**
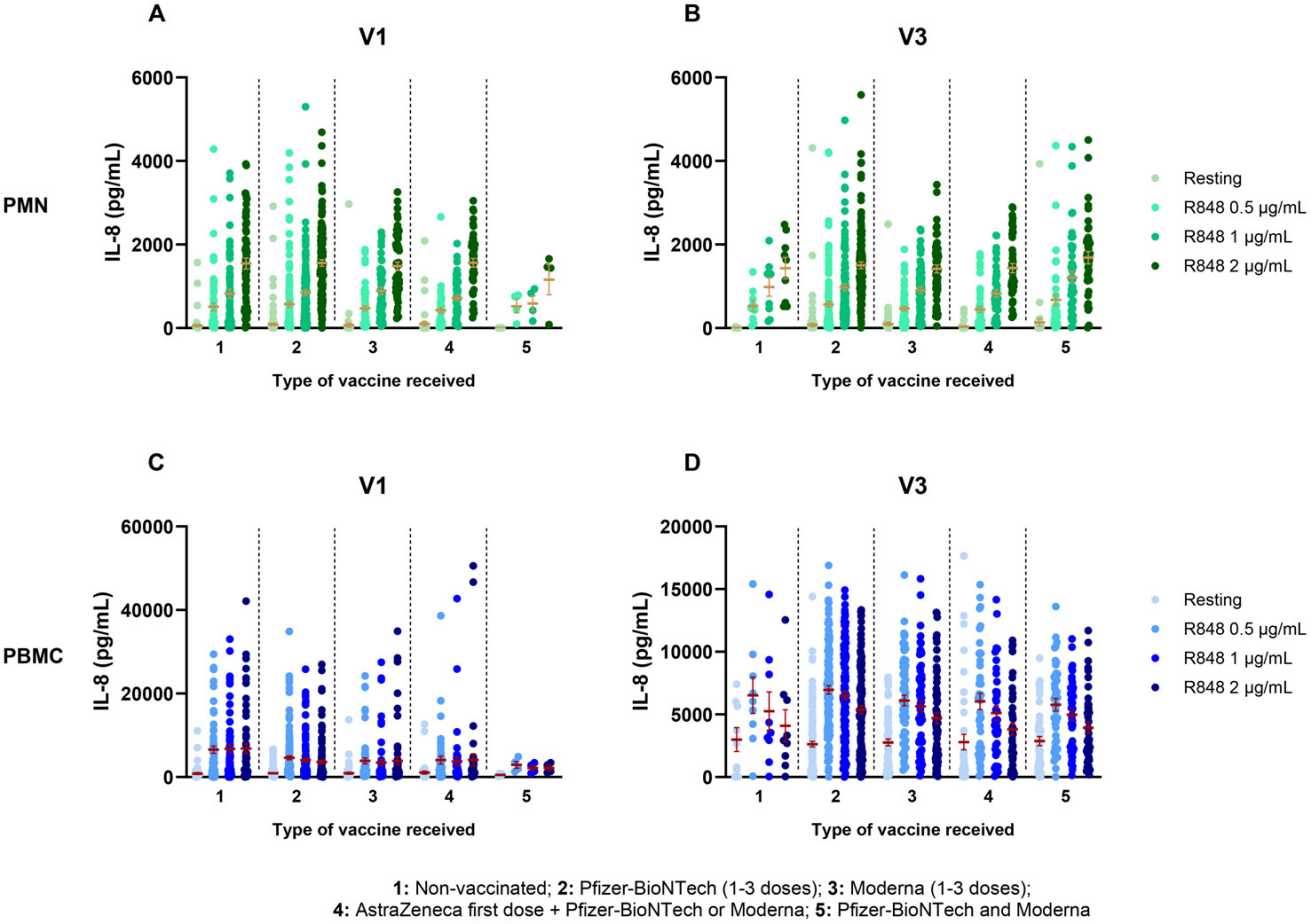
Il-8 response following activation by R848 separated by the type of vaccine received. **(A)** PMN IL-8 response at V1 (Group 1 n=65; group 2 n=129; group 3 n=59; group 4 n=46; group 5 n=4). **(B)** PMN IL-8 response at V3 (Group 1 n=9; group 2 n=128; group 3 n=63; group 4 n=44; group 5 n=43). **(C)** PBMC IL-8 responses at V1 (Group 1 n=64; group 2 n=129; group 3 n=60; group 4 n=46; group 5 n=4) **(D)** PBMC IL-8 responses at V3 (Group 1 n=9; group 2 n=129; group 3 n=65; group 4 n=44; group 5 n=43). Data shown are mean ± SEM. Kruskal-Wallis test with Dunn’s multiple comparisons test was used.

Depending on the type of vaccine received, participants were separated into five different groups. Group 1 consists of subjects who were not vaccinated. In groups 2 and 3, subjects received the BNT162b2 Pfizer-BioNTech vaccine (1-3 doses) or the mRNA-1273 Moderna vaccine (1-3 doses), respectively. Group 4 includes subjects who received the ChAdOx1-S AstraZeneca vaccine as a first dose, followed by either a second AstraZeneca dose, and/or one or two doses of an mRNA vaccine (Pfizer-BioNTech or Moderna). Finally, group 5 consists of participants who received a mix of mRNA vaccines (Pfizer-BioNTech and Moderna). Comparison of IL-8 production between those groups shows no effect of the type of vaccine regimen received on PMNs at V1, nor at V3 (**Figures 5A, B**). Likewise, no effect was seen for PBMC response at both visits (**Figures 5C, D**).

**Figure 6:**
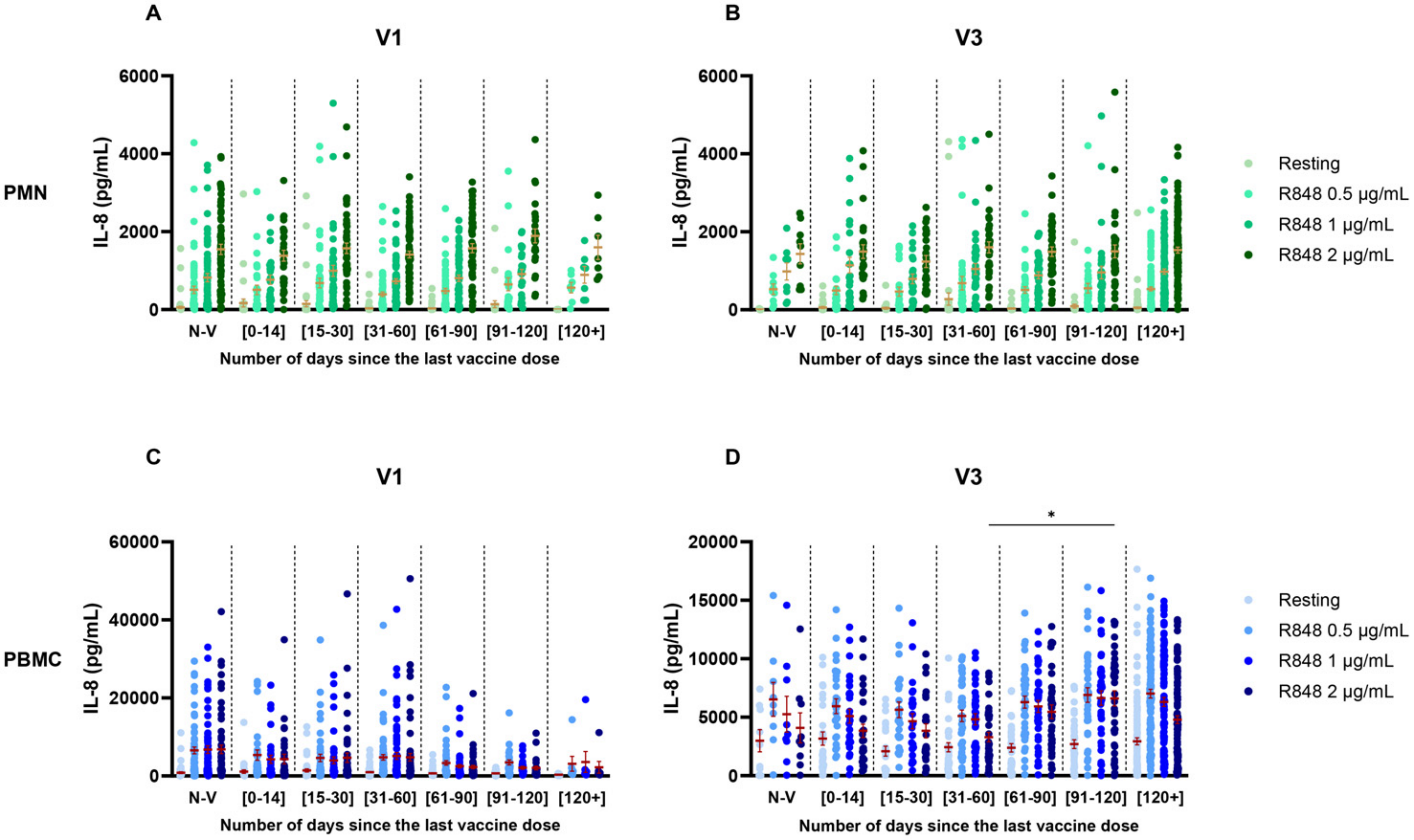
IL-8 response following R848 stimulation separated by the number of days between the visit and the last dose of vaccine received. **(A)** PMN response at V1 (Non-vaccinated n=65; [0-14] n=31; [15-30] n=48; [31-60] n=72; [61-90] n=55; [91-120] n=25; [120+] n=7). **(B)** PMN response at V3 (Non-vaccinated n=9; [0-14] n=27; [15-30] n=24; [31-60] n=36; [61-90] n=36; [91-120] n=35; [120+] n=120). **(C)** PBMC response at V1 (Non-vaccinated n=64; [0-14] n=31; [15-30] n=48; [31-60] n=72; [61-90] n=55; [91-120] n=26; [120+] n=7). **(D)** PBMC response at V3 (Non-vaccinated n=9; [0-14] n=28; [15-30] n=24; [31-60] n=36; [61-90] n=37; [91-120] n=37; [120+] n=119). Data shown are mean ± SEM. Kruskal-Wallis test with Dunn’s multiple comparisons test was used. * p <0.05.

Considering the variation in time between the last vaccine dose and the visits of each participant, additional analyses were performed to investigate if it influenced the IL-8 response. Groups were separated as follows: unvaccinated participants, last vaccine received less than 14 days, between 15 and 30 days, between 31 and 60 days, between 61 and 90 days, between 91 and 120 days, and more than 120 days prior to the visit. No effect was seen on the IL-8 response by PMNs at V1 and at V3 (**Figures 6A, B**). For the PBMC response, there was also no effect at V1 (**Figure 6C**). However, at V3 (**Figure 6D**) there was a significant increase in the IL-8 response following R848 stimulation (2 µg/mL) between subjects who received their last vaccine dose 31-60 days prior to their visit and those who received it 91-120 days prior to their visit.

**Figure 7:**
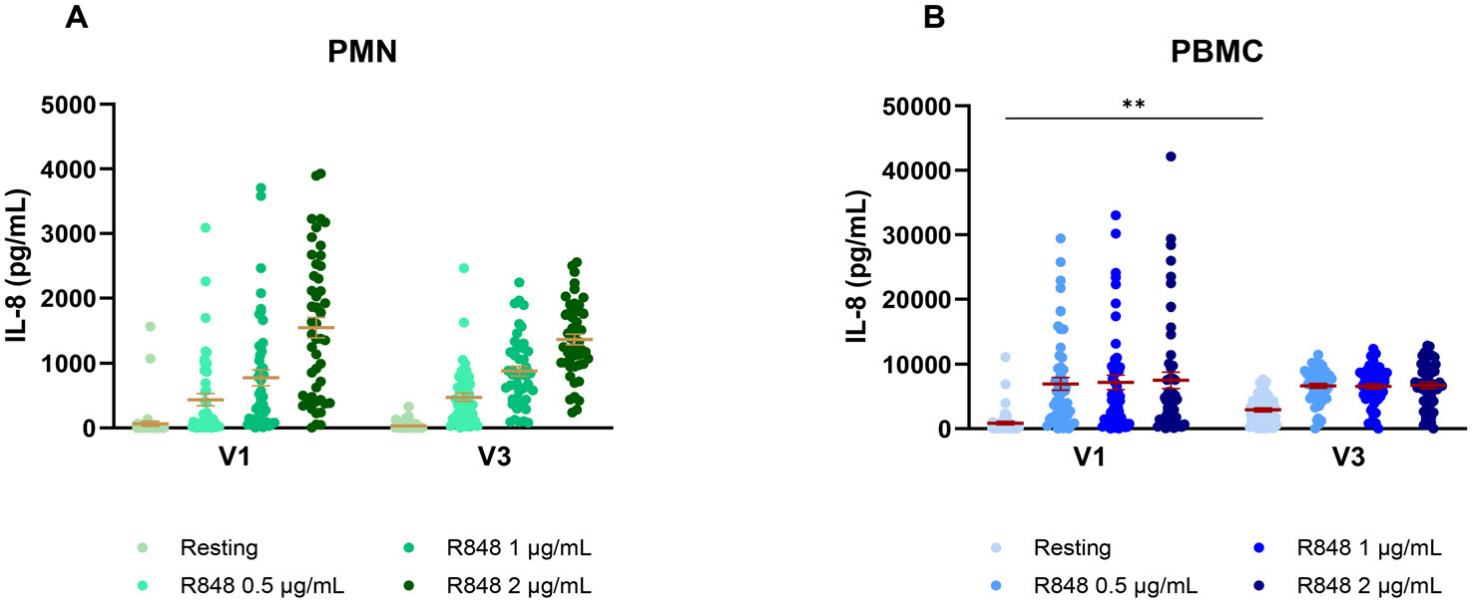
IL-8 response following R848 stimulation before and after COVID-19 vaccination. **(A)** IL-8 response by PMNs (n=48). **(B)** IL-8 response by PBMCs (n=51). Data shown are mean ± SEM. Friedman test with Dunn’s multiple comparisons test was used. ** p < 0.01.

This cohort also includes subjects who were not vaccinated against COVID-19 at the time of their first visit, who became fully vaccinated before the third visit. Hence, their IL-8 response over time was analyzed to see if vaccination had an impact or not. The PMN response remained unchanged between both visits (**Figure 7A**). However, the PBMC response without stimulation was significantly stronger at V3 compared to pre-vaccination (**Figure 7B**).

### 3.4 Seasonal effects on the inflammatory response

**Figure 8:**
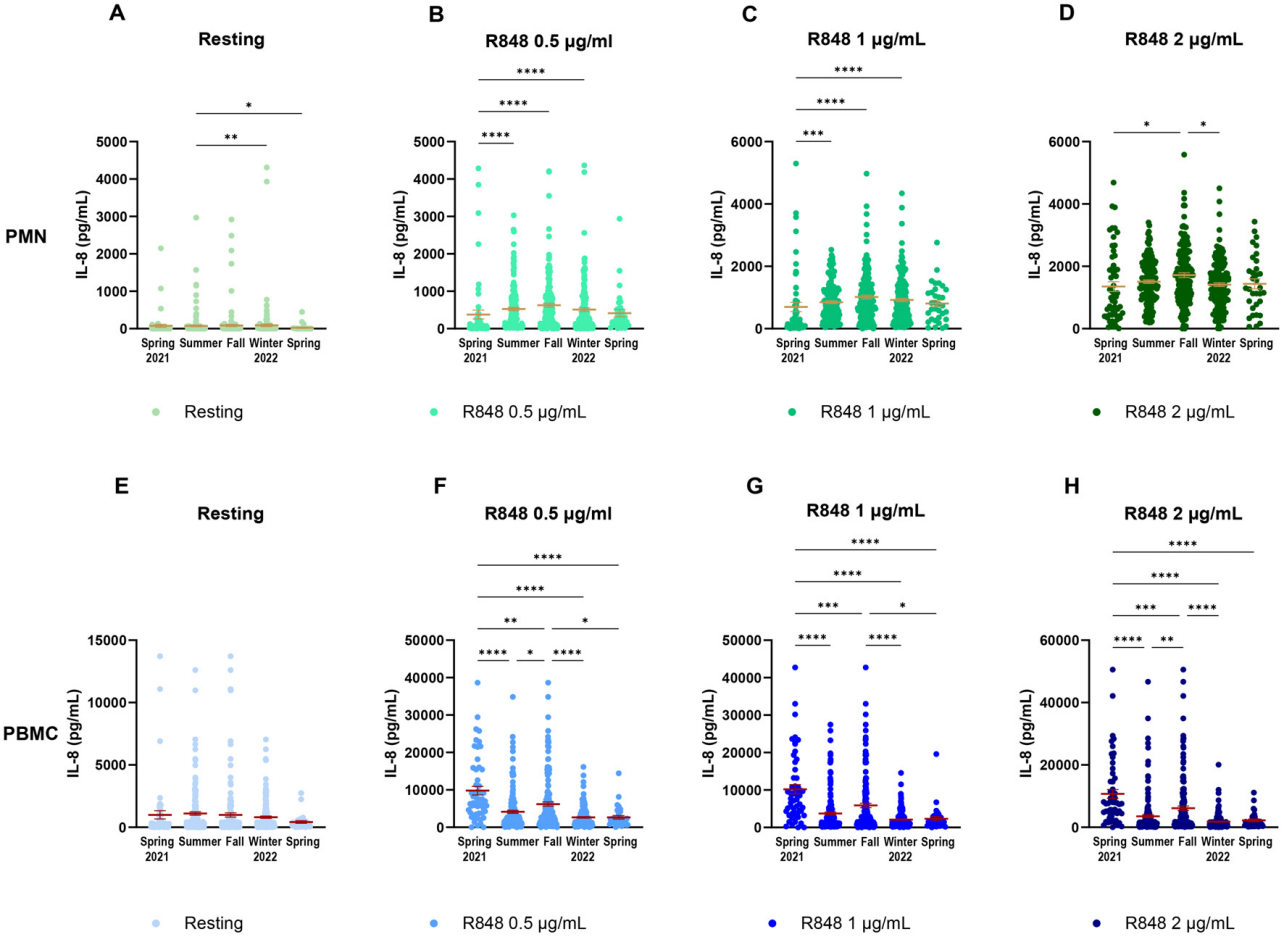
IL-8 response of PMNs and PBMCs to different concentrations of R848 stimulation separated by season of visit. **(A)** IL-8 response by PMNs at rest. **(B)** IL-8 response by PMNs after stimulation by 0.5µg/mL R848. **(C)** IL-8 response by PMNs after stimulation by 1µg/mL R848. **(D)** IL-8 response by PMNs after stimulation by 2µg/mL R848. **(E)** IL-8 response by PBMCs at rest. **(F)** IL-8 response by PBMCs after stimulation by 0.5µg/mL R848. **(G)** IL-8 response by PBMCs after stimulation by 1µg/mL R848. **H**) IL-8 response by PBMCs after stimulation by 2µg/mL R848. PMNs: Spring 2021 n=56; summer n=169; fall n=160; winter 2022 n=172; spring n=33. PBMCs: Spring 2021 n=55; summer n=169; fall n=163; winter 2022 n=173; spring n=33. Data shown are mean ± SEM. Kruskal-Wallis test with Dunn’s multiple comparisons test was used. * p < 0.05; ** p < 0.01; *** p < 0.001; **** p < 0.0001.

It is well known that the circadian rhythm affects the immune response (45, 46). Hence, the daily time of sampling was analyzed to investigate if it could influence our results (**Supplementary Figure S1**). Neither the PMN, nor the PBMC response to R848 stimulation was affected by the time at which samples were collected. As the study spanned over a whole year, and since the rolling averages showed some fluctuations, it seemed pertinent to evaluate seasonality on IL-8 responses. Each condition of stimulation was first separated and compared for the IL-8 response at each season of the study. For the PMN response, the resting condition (**Figure 8A**) is slightly increased in summer. Activation conditions (**Figures 8B–D**) display significant differences between spring 2021 and the following seasons. It is important to note that most participants who came during spring 2021 were not yet vaccinated against SARS-CoV-2. There is also a significantly higher IL-8 response after 2 µg/mL R848 stimulation in fall compared to winter. Responses during other seasons were similar. The PBMC response also shows strong variations between the different seasons of the study. Contrary to PMNs, the resting response remained stable throughout the course of the study (**Figure 8E**). The response after R848 stimulation also showed differences for PBMCs. Indeed, responses in spring 2021 were significantly stronger than in other seasons, as well as IL-8 responses in fall (**Figures 8F–H**). Moreover, differences remain marked at a high concentration of R848, contrary to PMNs. Variations in the responses separated by seasons do not concord with those following vaccination. Therefore, differences seen in PBMC response between seasons might be independent to the status of vaccination.

**Figure 9:**
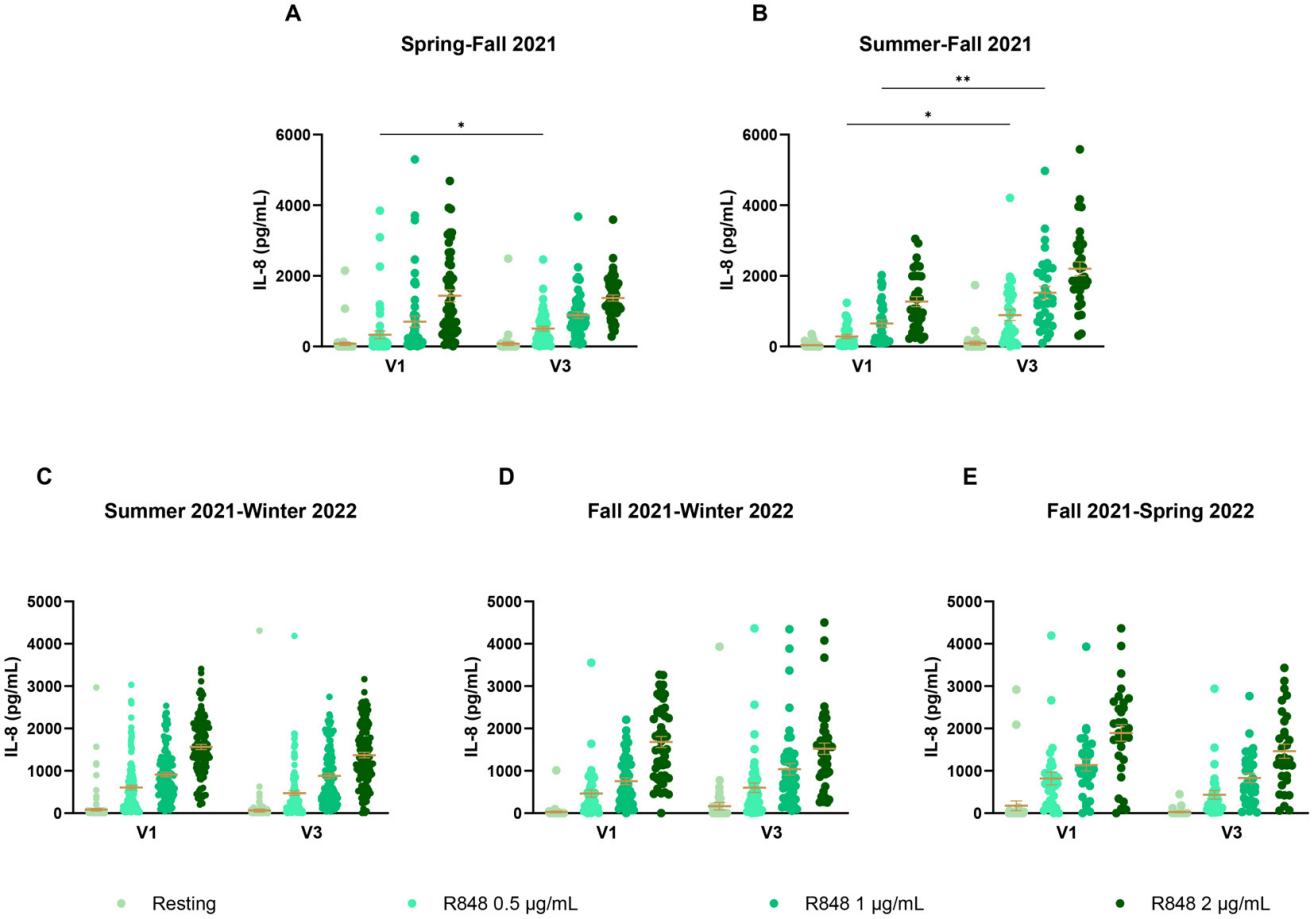
IL-8 response of PMNs following R848 stimulation compared between both visits of a same participant. **(A)** PMN IL-8 responses of participants who had their visit in spring and third in fall (n=49). **(B)** IL-8 responses by PMNs for participants who came in summer then in fall (n=33). **(C)** IL-8 responses by PMNs of participants who came in summer for the first visit and in winter for the third visit (n=125). **(D)** PMN IL-8 responses of subjects whose visits were in fall and winter (n=47). **(E)** PMN IL-8 responses of subjects who came in fall then in the following spring (n=31). Data shown are mean ± SEM. Friedman test with Dunn’s multiple comparisons test was used. * p < 0.05; ** p < 0.01.

**Figure 10:**
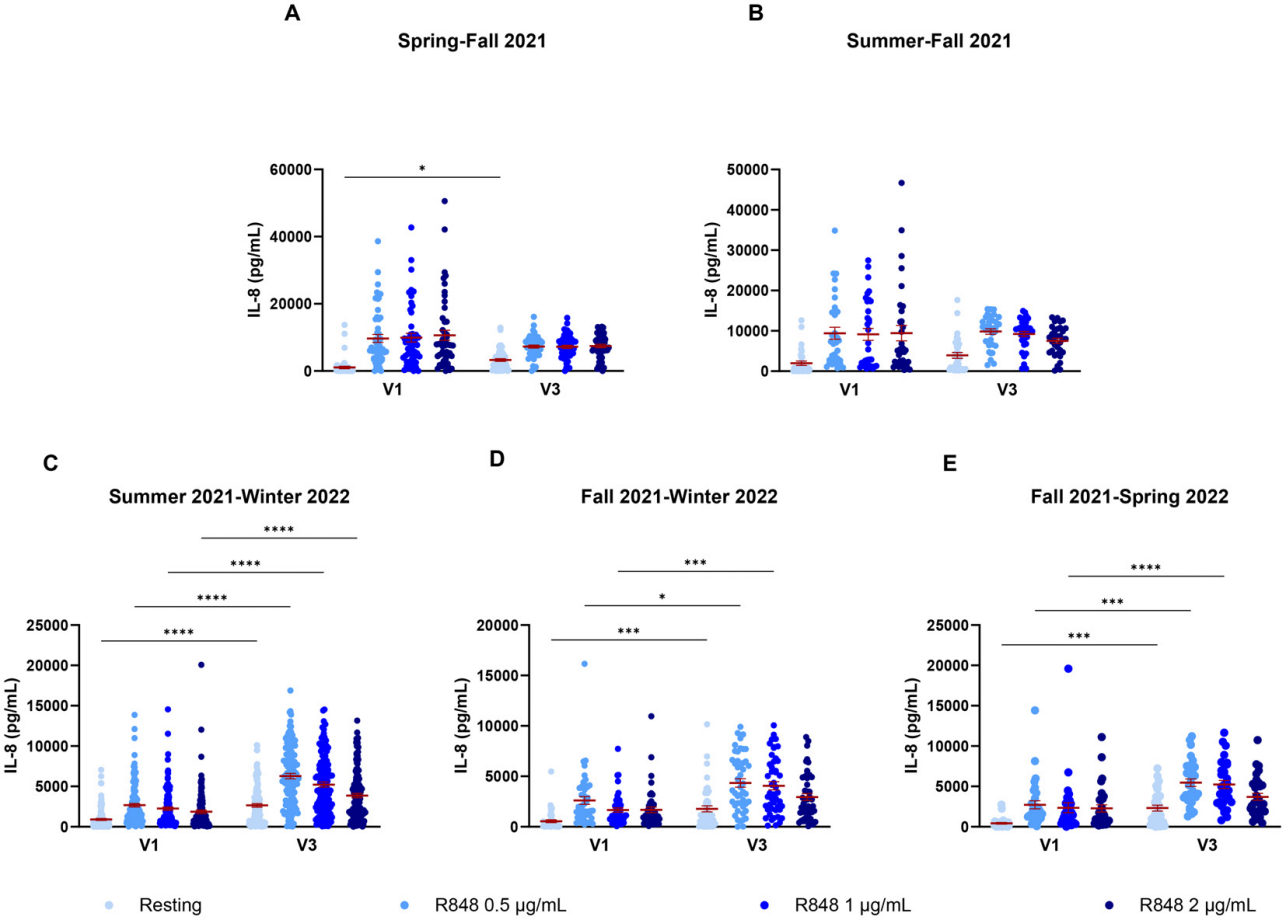
IL-8 response of PBMCs following R848 stimulation compared between both visits of a same participant. **(A)** PBMC response of subjects who came in spring and fall (n=51). **(B)** IL-8 response by PBMCs for participants who came in summer and fall (n=33). **(C)** PBMC response of subjects who had their first visit in summer and third in winter (n=126). **(D)** PBMC response for participants who came in fall and winter (n=47). **(E)** IL-8 response of subject’s PBMCs who had their visits in fall and spring (n=32). Data shown are mean ± SEM. Friedman test with Dunn’s multiple comparisons test was used. * p < 0.05; *** p < 0.001; **** p < 0.0001.

Since these results did not seem conclusive, seasonal differences were further investigated. PMN and PBMC activity was thus analyzed by comparing paired responses between visits 1 and 3 of participants, separating them by the seasons during which those visits took place. For PMNs (**Figure 9**), the strongest differences occur between summer and fall (**Figure 9B**), where there is an increase in the IL-8 response following stimulation in fall. Interestingly, PMN activation also increases in fall compared to spring (**Figure 9A**). Here, the majority of participants were not vaccinated in spring for their first visit, but received their full vaccine before their third visit. This result differs from those seen earlier in **Figure 7A**, suggesting that the season might have a greater impact than vaccination on PMN responses. No difference was observed for other seasons (**Figures 9C–E**). As no participants came first in spring and then in summer for their third visit, that comparison could not be made. For PBMCs (**Figure 10**), stronger responses were seen between summer and winter, as well as between fall and winter and between fall and spring. Surprisingly, all significant differences were an increase in the PBMC response at V3, no matter the season, suggesting that the changes seen between seasons might be due to other factors. There was an increase of IL-8 production for PBMCs at rest between spring and fall (**Figure 10A**), but no differences between summer and fall (**Figure 10B**). In subsequent analyses, IL-8 measurements were separated by month. It was found that there were many fluctuations during the year. For the PMN response, there seem to be peaks of activity across summer and fall following R848 stimulation (**Supplementary Figure S2**). The PBMC response shows strong peaks at the end of spring and at the end of fall (**Supplementary Figure S3**). The first peak coincides with the first wave of vaccination, as seen in the timeline (**Figure 1B**). However, the second peak comes before peak vaccination and the burst of infections observed during the fifth wave, which indicates that it must be independent of vaccination or infection.

**Figure 11:**
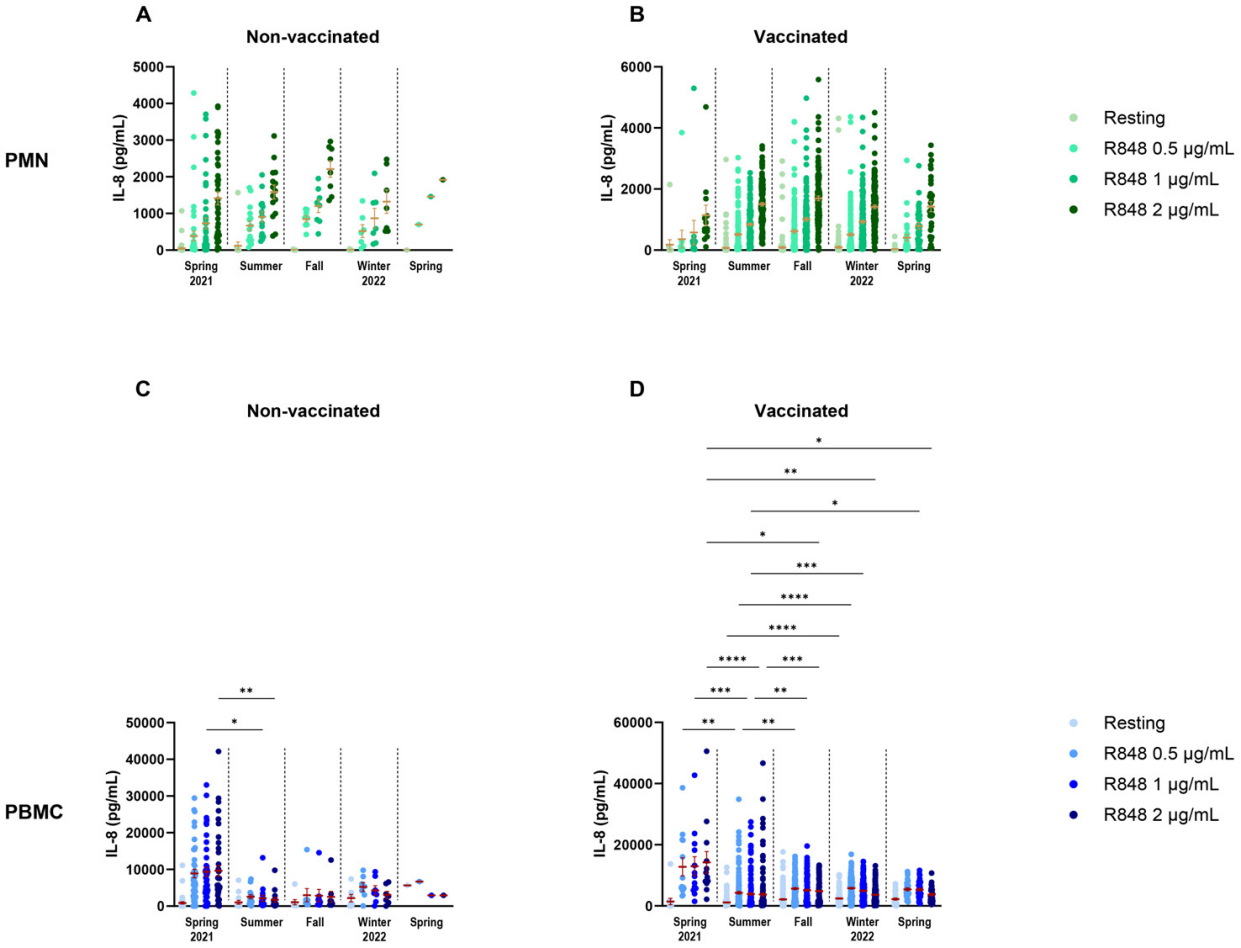
Comparison of IL-8 response following R848 stimulation in relation to seasons and vaccination status. **(A)** Non-vaccinated subjects’ PMN responses by season of visit (spring 2021 n=43; summer n=15; fall n=8; winter 2022 n=7; spring n=1). **(B)** Vaccinated participants’ PMN IL-8 responses by season of visit (spring 2021 n=13; summer n=154; fall n=152; winter 2022 n=165; spring n=32). **(C)** Non-vaccinated subjects’ PBMC responses by season of visit (spring 2021 n=42; summer n=15; fall n=8; winter 2022 n=7; spring n=1). **(D)** Vaccinated subjects’ PBMC IL-8 responses by season of visit (spring 2021 n=13; summer n=154; fall n=156; winter 2022 n=166; spring n=31). Data shown are mean ± SEM. Kruskal-Wallis test with Dunn’s multiple comparisons test was used. * p < 0.05; ** p < 0.01; *** p < 0.001; **** p < 0.0001.

**Figure 12:**
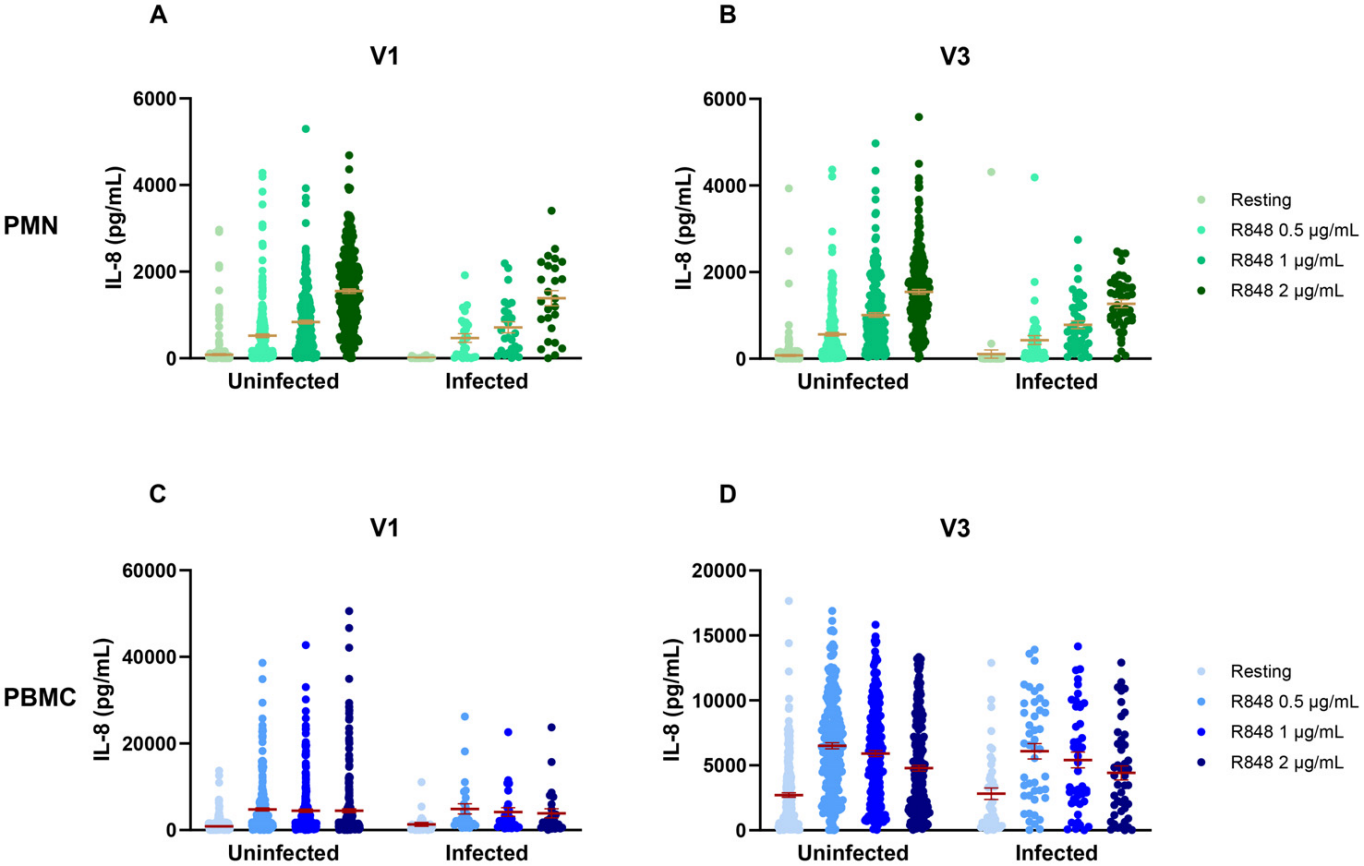
IL-8 response to R848 stimulation in COVID-19 uninfected and infected participants. **(A)** IL-8 response by PMNs at the first visit (uninfected n=276, infected n=26). **(B)** IL-8 response by PMNs at the third visit (uninfected n=277, infected n=26). **(C)** IL-8 response by PBMCs at the first visit (uninfected n=240, infected n=45). **(D)** IL-8 response by PBMCs at the third visit (uninfected n=244, infected n=45). Data shown are mean ± SEM. Kruskal-Wallis test was used with Dunn’s multiple comparisons test.

To attempt to discriminate further between seasons and vaccination, vaccinated and non-vaccinated participants were separated in the seasonal analysis. For PMNs, there were no differences in IL-8 between seasons for both non-vaccinated and vaccinated participants (**Figures 11A, B**). It is notable that, except for spring 2021, the number of non-vaccinated subjects who came during following seasons was rather small (summer n=15, fall n=8, winter n=7, spring 2022 n=1); hence, it is difficult to draw conclusions for those groups. The results for PBMCs were quite different. Indeed, there was only a difference between spring and summer 2021 in the IL-8 response following activation with 1 and 2 µg/mL R848 for the non-vaccinated participants (**Figure 11C**). In contrast, vaccinated participants showed differences for all conditions of activation (**Figure 11D**). Finally, analyses were done to compare IL-8 production in COVID-19 infected and uninfected participants (**Figure 12**) and no differences were seen between both groups.

## 4 Discussion

In this study, the role of SARS-CoV-2 vaccination and the effect of seasonality relative to the innate immune response were investigated. Indeed, innate immunity is key to supporting adaptive immunity, which is much more effective in combatting pathogens (47). If the innate response fluctuates based on external changes, the overall immune response following infection could also be impacted. This relates to the concept of trained immunity that stipulates that, upon primary stimulation with a pathogen, the innate immune response undergoes certain epigenetic changes that allow it to react with more readiness following a secondary stimulation by the same or different pathogens (48–50). It is known that, not only infections, but vaccines have the potential to induce trained immunity (49, 51–54). For example, several groups have studied the impact of the bacillus Calmette-Guerin (BCG) vaccine on the immune response to COVID-19 infection at the start of the pandemic, as it is a well-known mediator of trained immunity, and have indeed found a protective effect (38, 55–59). Recent data also found that severe COVID-19 induces innate memory (60). Since the approval of COVID-19 vaccines, a few studies have also found that they induced trained immunity (61–63). Others, however, show that, although there was a stronger innate response following vaccination, those alterations were not significant in the long-term (64, 65). In this study, the IL-8 response of PMNs and PBMCs was evaluated looking at different vaccinal parameters. Only the PBMC response was affected by vaccination, with a significant decrease in the IL-8 response to R848 stimulation after 2 or 3 vaccine doses. When correlating time between a visit and the last vaccination, there was only a small window of time during which PBMC responses were increased, consistent with the previous studies (63, 64). This is also in agreement with previous research showing that vaccine immunity decreased overtime, explaining the need for booster doses (31, 66–68). The decrease in IL-8 response following vaccination could also be due to the desensitization of TLRs caused by a previous activation. In fact, previous works have shown that repeated TLR stimulation by an infection or a synthetic compound like R848 can decrease a subsequent response (69, 70).

In this study, immune responses seemed to change with the seasons. This was more marked in the PBMC response, for which peaks in IL-8 production can be seen at the end of spring and at the end of fall. It is well known that some infections follow seasonal cycles, such as influenza (40, 71). In fact, the innate antiviral defense can be affected by different environmental factors that vary across seasons, such as temperature and humidity (72–74). More specifically, colder temperatures decrease the induction of type I IFN in infected cells (72). This is significant since type I IFN have a major role to play in antiviral immune responses (8). More importantly, type I IFN have been found to play an inhibitory role on IL-8 production in PBMCs, but not in PMNs (75). IFN-stimulated genes have been found to be upregulated following R848 stimulation in monocytes, but not in neutrophils (18). We found a clear variation in PBMCs’ IL-8 production throughout the year, but not so much for PMNs. This suggests that type I IFN, influenced by environmental factors, could play a role in the IL-8 response seen in this cohort. Indeed, there was an increase in IL-8 during colder months (November to January), which could be hypothesized to be caused by a decrease in type I IFN due to cold temperatures. This study may not determine the source of cytokine expression among the IL-8 producing cells upon R848 stimulation, such as dendritic cells (DCs), monocytes/macrophages, and natural killer (NK) cells in PBMCs (18, 76–78). Exhaustive phenotypic and transcriptomic analyses could be done to answer this question and the dynamic of cellular activation.

The cohort was established to assess SARS-CoV-2 exposure among essential workers in Quebec by analyzing the infection rate and identifying risk factors based on their demographic, occupational, and residential characteristics (42) and measuring seroprevalence using three methods: pseudo-neutralization (79), antibodies measurement by ELISA (80), and live microneutralization (81). The study presented here is part of this large group project and aims to evaluate innate immune responses. Interestingly, correlations can be established between other measures of humoral immunity taken from the cohort. Indeed, as there was an increase in PBMCs’ IL-8 response in May-June 2021, the pseudo-neutralization assay showed an increase in neutralizing antibody titers around July-August 2021 (79). The first period corresponds to the first wave of COVID-19 vaccination, while July-August is when the majority of subjects had received two doses of vaccine. In both cases, the response declined thereafter, and peaked again around November-December 2021 for IL-8 and in January 2022 for neutralizing antibodies. These periods correspond to the start of the Omicron wave during which many subjects were infected, as well as to the vaccination campaign for the third dose.

Regarding the live microneutralization assay, it showed that neutralizing antibody titers in the serum were significantly lower in subjects who received only two doses, compared to those who received three doses (81). It contrasts with the IL-8 response, measured in this paper for PBMCs that shows a significant decrease with an increase in vaccine doses. Although neutralizing antibody titers are increased upon receiving a booster dose of vaccine, the changes taking place in innate immunity are decreasing PBMC activation following stimulation.

One limitation of this study concerns its design, as it initially planned to evaluate the seroprevalence. The rapid implementation of the vaccination campaign and great rate of participation have changed the study. In fact, when subjects started being recruited, the vaccination campaign was also just taking off in the province, with 27% of the population having already received at least one dose, and more than 83% of the population being fully vaccinated at the end of the study period in May 2022 (82). Therefore, there is a small number of non-vaccinated subjects as the recruitment was meant to be representative of the general population working in food and retail stores.

In conclusion, this study highlights the importance of considering confounding factors when studying innate immunity. Indeed, we have shown that vaccination influences the PBMC response following activation and that seasonality influences PMN and PBMC responses differently. Furthermore, we highlight the importance of implementing a more rigorous matching strategy that considers the season of sample collection, and of grouping participants based on comparable seasons to better distinguish between intrinsic immune responses and those influenced by vaccination. This approach will help reduce potential biases related to seasonality and strengthen the validity of analyses. In the long term, it will be relevant to conduct a new prospective longitudinal study with monthly measurements, including a control group of unvaccinated participants matched based on relevant characteristics (e.g., age, gender, health status) and environmental data to firstly compare innate immune responses in healthy and vulnerable people.

## Data Availability

The original contributions presented in the study are included in the article/**Supplementary Material**. Further inquiries can be directed to the corresponding author.

## Supplementary material

Supplementary Figure 1 IL-8 response following R848 stimulation compared by time of blood sampling. **(A)** PMN responses at V1 (Morning n=163; afternoon n=140). **(B)** PMN responses at V3 (Morning n=156; afternoon n=131). **(C)** PBMC responses at V1 (Morning n=162; afternoon n=141). **(D)** PBMC responses at V3 (Morning n=157; afternoon n=133). Data shown are mean ± SEM. Kruskal-Wallis test with Dunn’s multiple comparisons test was used.

Supplementary Figure 2 IL-8 response separated by month of visit for stimulation of PMNs with R848. **(A)** IL-8 production at rest. **(B)** IL-8 response to 0.5 µg/mL R848 stimulation. **(C)** IL-8 response to 1 µg/mL R848 stimulation. **(D)** IL-8 production after 2 µg/mL R848 stimulation. Data shown are mean ± SEM.

Supplementary Figure 3 IL-8 response separated by month of visit for stimulation of PBMCs with R848. **(A)** IL-8 production at rest. **(B)** IL-8 response to 0.5 µg/mL R848 stimulation. **(C)** IL-8 response to 1 µg/mL R848 stimulation. **(D)** IL-8 production after 2 µg/mL R848 stimulation. Data shown are mean ± SEM.
