# Supplementary material for: Impact of SARS-CoV-2 vaccination and of seasonal variations on the innate immune inflammatory response
Source: Front Immunol. 2025 Jan 14;15:1513717. doi: 10.3389/fimmu.2024.1513717 (PMC11772892; doi:10.3389/fimmu.2024.1513717)
Supplement: Supplementary Figure 1 — IL-8 response following R848 stimulation compared by time of blood sampling. (A) PMN responses at V1 (Morning n=163; afternoon n=140). (B) PMN responses at V3 (Morning n=156; afternoon n=131). (C) PBMC responses at V1 (Morning n=162; afternoon n=141). (D) PBMC responses at V3 (Morning n=157; afternoon n=133). Data shown are mean ± SEM. Kruskal-Wallis test with Dunn’s multiple comparisons test was used. [file DataSheet1.pdf]

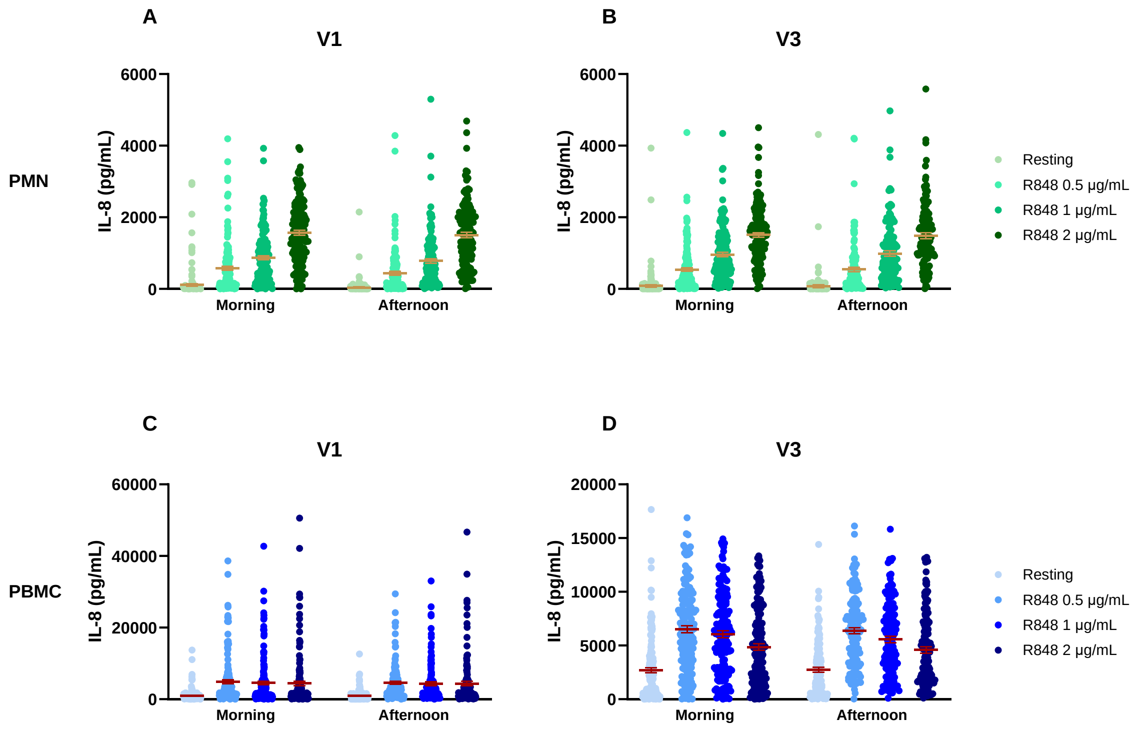

Supplementary figure 1. IL-8 response following R848 stimulation compared by time of blood sampling. A) PMN responses at V1 (Morning n=163; afternoon n=140). **B)** PMN responses at V3 (Morning n=156; afternoon n=131). **C)** PBMC responses at V1 (Morning n=162; afternoon n=141). **D)** PBMC responses at V3 (Morning n=157; afternoon n=133). Data shown are mean ± SEM. Kruskal-Wallis test with Dunn's multiple comparisons test was used.

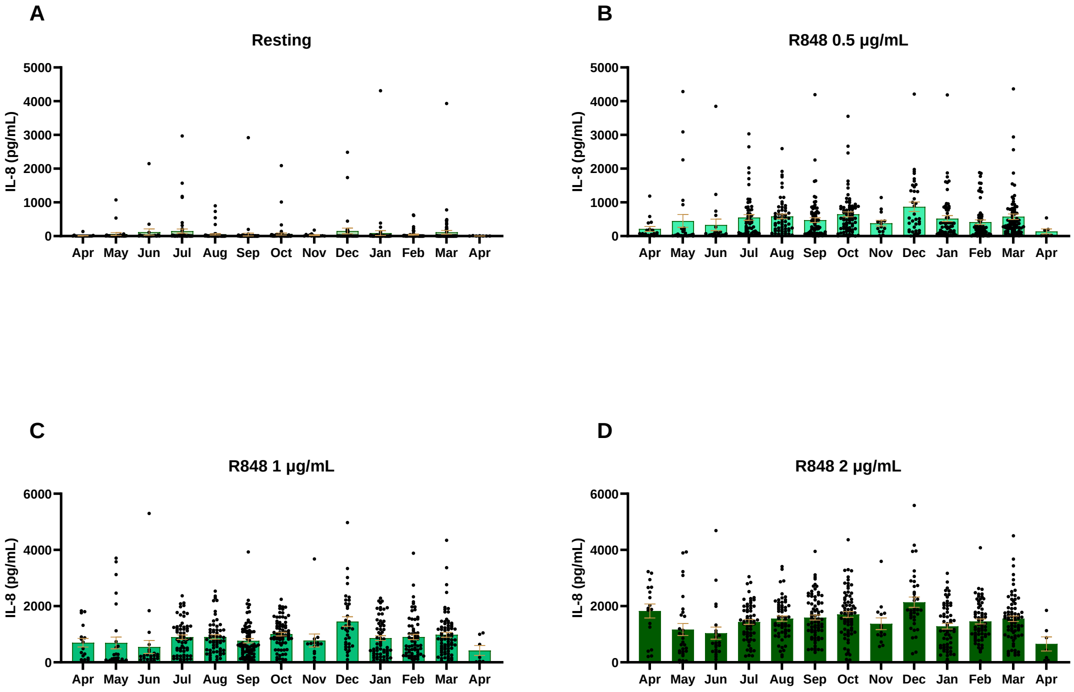

Supplementary figure 2. IL-8 response separated by month of visit for stimulation of PMNs with R848. A) IL-8 production at rest. **B)** IL-8 response to 0.5 µg/mL R848 stimulation. **C)** IL-8 response to 1 µg/mL R848 stimulation. **D)** IL-8 production after 2 µg/mL R848 stimulation. Data shown are mean ± SEM.

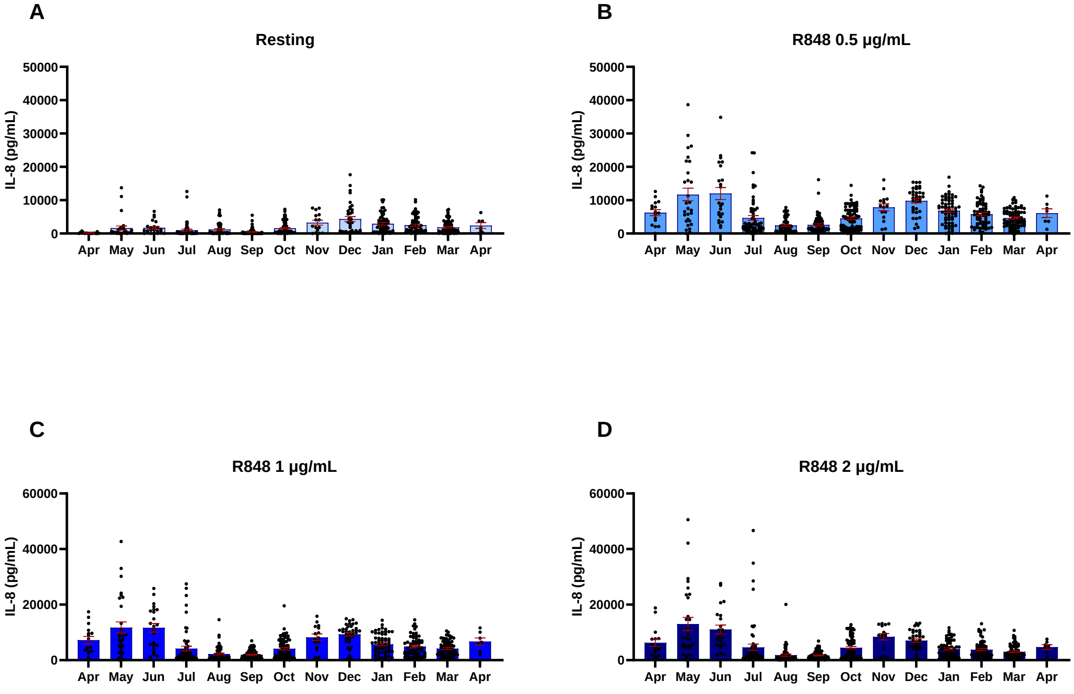

Supplementary figure 3. IL-8 response separated by month of visit for stimulation of PBMCs with R848. A) IL-8 production at rest. **B)** IL-8 response to 0.5 μg/mL R848 stimulation. **C)** IL-8 response to 1 μg/mL R848 stimulation. **D)** IL-8 production after 2 μg/mL R848 stimulation. Data shown are mean ± SEM.
